# The Associations Between Parental Playfulness, Parenting Styles, the Coparenting Relationship and Child Playfulness

**DOI:** 10.3390/bs15070867

**Published:** 2025-06-26

**Authors:** Harshita Seal, Jean-François Bureau, Audrey-Ann Deneault

**Affiliations:** 1 School of Psychology, University of Ottawa, 136 Jean-Jacques-Lussier Private, Ottawa, ON K1N 6N5, Canada; 2 Département de Psychologie, Université de Montréal, 90 Vincent-d’Indy Avenue, Montréal, QC H2V 2S9, Canada; audrey-ann.deneault@umontreal.ca

**Keywords:** parental playfulness, parenting, coparenting relationship, child playfulness, fatherhood

## Abstract

This study explores the associations between parental playfulness and various aspects of parenting, specifically role overload, parenting behaviors, and the quality of coparenting. In addition, we explore the relation between parental playfulness and child playfulness, as well as the differences in playfulness between mothers and fathers. This cross-sectional, questionnaire-based study relied on a sample of 348 parents (84.7% mothers) of children aged 3–8 years old (52.9% girls). Significant associations were found between parental playfulness and cognitive aspects of child playfulness (e.g., sense of humor). Certain dimensions of coparenting (e.g., support) and other parenting behaviors (e.g., challenging and warmth) were also correlated with parental playfulness. There was no association found between parental playfulness and parental role overload. However, a significant moderation effect of parental gender was found only for the association between parental playfulness and role overload. This analysis showed that parental playfulness was positively associated with role overload for fathers but was negatively associated for mothers. Lastly, we did not identify differences between mothers’ and fathers’ self-reported playfulness. These results provide important information about an understudied parental behavior, which may inform interventions promoting positive parenting.

## 1. Introduction

Parenting styles and behaviors, including their level of sensitivity or favorite method of play, can have profound effects on the development of children’s socioemotional and cognitive development (e.g., Cooke et al., 2022; Madigan et al., 2019; Rodrigues et al., 2021; Stgeorge & Freeman, 2017). An abundance of researchers has focused on how to improve parenting by remedying negative attributes without examining positive traits that could be strengthened. Amongst positive parental behaviors that are frequently studied is a parent’s ability to foster secure child–parent attachments through sensitive caregiving, especially in moments of distress (Jones et al., 2015). Despite the demonstrated importance of sensitive caregiving, it is essential to investigate the other positive aspects of parenting. Particularly, it is important to consider whether parents enjoy their role as a parent (Aytuglu & Brown, 2022) and how parents interact with their children when they are not in dire need of assistance or support (Bureau et al., 2014). A child who is cared for by someone who enjoys parenthood may feel more valued and as if they deserve the parental attention and affection they receive voluntarily.

In the current study, we focus specifically on parental playfulness. Parental playfulness is defined as a parent’s use of creativity, imagination, and humor during parent–child interactions (Menashe-Grinberg & Atzaba-Poria, 2017). This type of playfulness is specific to behaviors that emerge within the parent–child relationship, without generalizing it to other areas of a parent’s life. Parental playfulness can be characterized by positive affect, such as shared enjoyment and genuine laughter; it may also involve cognitive components, including creativity and perspective-taking. This construct can be distinct from trait playfulness, which is more reflective of a predisposition to approach life with spontaneity and humor across a variety of situations (Glynn & Webster, 1992). While these concepts may be related, they may be expressed differently, as some parents may find it easier to be playful with their children than with other adults, and some may voluntarily make an effort to be playful with their child because they view playfulness as important for their children’s development. The subject of parental playfulness is quite novel, thus making it challenging to know how this behavior compares to other parental behaviors. For example, Majdandžić et al. (2016) found that fathers who encourage their children to explore outside of their comfort zone and engage in challenges tend to be warmer, which is not something that is typically expected based on the more traditional literature on maternal sensitivity. This exemplifies how much we still do not know about parental playfulness and the type of parent that exhibits it.

The main objective of this study was to explore the associations between parental playfulness and other aspects of parenting. Specifically, we examined whether parental playfulness is associated with parental role overload (i.e., how overwhelmed one feels with their role as a parent), parental involvement, the endorsement of various parenting behaviors (e.g., expressing affection physically, disciplining their children, or encouraging their children to engage in risk-taking activities), and the quality of the coparenting relationship. We acknowledge that parents may model certain behaviors which can be observed and imitated by children which, in turn, can affect their development. Therefore, in addition to these parenting areas, we also found it important to consider the association between parental playfulness and child playfulness. Furthermore, considering the distinction between context-specific and trait playfulness, we would also like to include general adult playfulness in relation to the various parental domains.

### 1.1. Children’s Playfulness and Parental Playfulness

The literature indicates that playfulness is a beneficial trait for children to possess. Child playfulness is associated with emotional adjustment in children, such that playful children show more self-confidence, independence, curiosity, and imagination in their general social functioning (Barnett, 1991a). One study found a strong positive correlation between playfulness in children and their ability to cope with stress in their school environment (Saunders et al., 1999). This association may be due to the fact that coping behavior and effective play involve similar mechanisms, including creativity, persistence, active engagement, flexibility, and adaptability to each environment (Saunders et al., 1999). Additionally, playful children demonstrate more cooperativeness and positive affect, in general, than less playful children (Shorer et al., 2021).

As child playfulness seems to play an important role in their socioemotional functioning, it is important to better understand what factors could contribute to its development. One important contributor may be their parent’s own playfulness. In line with the social learning theory proposed by Bandura (1971), children learn from example; thus, it is possible that playful parents could elicit playfulness within their offspring. Youell (2008), in a series of observations supporting this theory, describes how young children learn to be playful by imitating behavior showcased by their caregiver. A literature review conducted by von Salisch (2001) examined articles that provided information surrounding how emotional expression is developed in children through their parents, peers, and friends. The author addresses evidence indicating that parents assist in emotional development through their cultural teachings of appropriate emotional responses and regulation (von Salisch, 2001). Applying this theory to the attribute of playfulness, parents may teach their children appropriate times in which to express humor or playfulness as well. von Salisch (2001) also found that children developed emotional expression through individual differences between parents in their emotional expression. For example, fathers who were found to be more emotionally expressive tended to have children who displayed a better understanding of emotions and were better liked than their peers (von Salisch, 2001). In this case, we can identify a connection between what is displayed by the parent and how it may, in turn, influence children’s behavior as well. Likewise, we are interested in investigating whether the trait of playfulness, like emotional expression, can be developed through parental traits as well. A recent multi-methods study investigated whether playful parents raise playful children (Wu et al., 2024). The authors surveyed 360 parents of children aged 4–6 years old in Singapore and found, through the use of questionnaire data, a significant positive correlation between parental playfulness and child playfulness (Wu et al., 2024). Additionally, they interviewed 10 parents from the quantitative sample, 5 of whom had children with high playfulness and 5 of whom had children with low playfulness. In the high playfulness group, the authors found that the parents who attached importance to their own play also actively encouraged their children to play; however, with the low playfulness group, the parents were less engaged and allowed their children to play independently as they wished (Wu et al., 2024). The information found by these authors suggests that parental traits, including playfulness, can possibly be transmitted, thus supporting a need to explore the construct of parental playfulness in association with child playfulness.

Parental playfulness not only affects the levels of playfulness in children but can also affect other attributes. Menashe-Grinberg and Atzaba-Poria (2017) found that parents who scored higher in levels of playfulness had children who displayed lower levels of negativity. Findings from Shorer et al. (2021) also indicated that more parental playfulness is associated with better emotional regulation in their children. Included in the realm of parental playfulness is teasing, which can be defined as “an interactional episode in which the parent behaves in a particular way in order to destabilize the infant by contradicting his ongoing action and his expectations” (Labrell, 1994, p. 126). Labrell (1994) went on to address the benefits of teasing, suggesting that it allows children to create coping strategies for conflict, how to deal with it, and adapt to the situations without any serious resentment towards the parent. Parental playfulness has been researched in association with child outcomes, however, no study has looked at this trait and its associations with other facets of parenting.

### 1.2. Parental Playfulness and Other Parenting Behaviors

A playful parent may feel more satisfied with their parenting role, but the reverse is also plausible: more satisfied parents may become more playful. An important characteristic linked to positive parental behaviors (e.g., more positive interactions with children, use of encouragement, and equal engagement in activities) is parental self-efficacy (Román-Oyola et al., 2017). Parental self-efficacy refers to the degree of confidence a parent has in how effective they perform in their role as a parent (Román-Oyola et al., 2017). The study conducted by Román-Oyola and colleagues found that adult playfulness was the most relevant predictor of emotional parental self-efficacy (Román-Oyola et al., 2017), suggesting that adult playfulness and healthy parenting behavior are related. Additionally, Majdandžić et al. (2016) conducted a study examining challenging parenting behaviors, overprotectiveness, and warmth. These authors define challenging parenting behaviors as the degree to which parents emotionally or physically encourage their child, in a playful way, to engage in risky behavior or behaviors outside of the child’s comfort zone. In this study, they found that parents who exhibited more challenging parenting behaviors were generally warmer, thus indicating that challenging parenting behavior could be a positive aspect of parenting. Seemingly, according to the traditional literature on maternal sensitivity, challenging behavior would not coincide with warmth, although the way in which these terms have been defined seems to indicate that promoting risky or unfamiliar behaviors (e.g., pillow fighting, chasing, and gentle wrestling) in children come from a place of care for their development. The behaviors may, thus, be accompanied by warm behaviors such as physical or verbal affection. This would also be in line with attachment theory, postulating that encouraging exploration in children when they are not distressed or in need of proximity is also part of parental sensitivity. As such, a parent acts as a secure base from which the child explores their physical and social environment (Hoffman et al., 2006). The direct link between parental playfulness and other parenting behaviors has not been investigated thoroughly, yet these studies appear to indicate a relation between these parental behaviors.

### 1.3. Parental Playfulness and Coparenting Relationships

Playfulness in adults has been shown to predict psychological well-being, life satisfaction, and life engagement (Proyer, 2013); however, we may also wonder how it may affect the coparenting relationship. Proyer (2014b) discusses how play or being playful can elicit positive emotions, which, according to Fredrickson (2001), can assist in building resources such as psychological resiliency that may aid in maintaining relationships. Furthermore, there is empirical evidence to suggest that positive emotions are positively associated with romantic relationship satisfaction (Aune & Wong, 2002; Baxter, 1992; Proyer, 2014b). Other research has indicated that adults who are playful are less stressed than their counterparts who are not playful (Shorer & Leibovich, 2022). These adults can use better stressor-focused coping strategies instead of employing negative, avoidant, or escape-oriented strategies (Shorer & Leibovich, 2022). Glynn and Webster (1992) assessed adult playfulness as a psychological trait and found that a playful person is associated with various positive outcomes in their workplace, possibly due to their abilities to defuse tension and build more positive social relationships. In the context of parenting, a playful parent may be able to reduce the tension associated with a disagreement regarding child-rearing, therefore fostering a better context for the search for a constructive solution.

With these studies in mind, the assumption would be that a playful partner would make a coparenting relationship stronger. On the other hand, the intimate nature of play could facilitate feelings of jealousy due to the child’s potential favoritism for the more playful caregiver, and/or feelings of being undermined by the other parent. A recent study by Bureau et al. (2023) showed that parents who feel excluded from their partner–child play rate their coparenting relationship more negatively. It is also possible that the unconventional and disruptive nature of playful parenting may be frowned upon by a partner who shows their disapproval. In studies conducted by McBride et al. (2005) and Anderson et al. (2017), they found that mothers’ parenting styles or attitudes had an impact on the quality of play or investment the father engaged in with their child, therefore suggesting the possibility that a coparent’s disapproval may reduce parental involvement in playful behaviors. This, in turn, could result in feelings of resentment. Due to the opposing perspectives on how coparenting may relate to parental playfulness, it is in our best interest to investigate this relationship.

### 1.4. Parental Playfulness and Role Overload

Parenting can be stressful and can pose some unexpected challenges, which may lead to feelings of being overburdened by one’s role as a parent. However, as mentioned previously, playfulness, in general, is associated with better coping strategies and less stress (Labrell, 1994; Saunders et al., 1999; Shorer & Leibovich, 2022). These strategies may potentially aid in alleviating these feelings. Barnett (2007) investigated which qualities characterize a playful individual and was able to define adult playfulness, based on their findings, as the “predisposition to frame (or reframe) a situation in such a way as to provide oneself (and possibly others) with amusement, humor, and/or entertainment” (p. 955). This ability to reframe a situation could allow individuals to experience fewer overburdening feelings as a parent. As such, playful parents may actually find enjoyment in their responsibilities rather than being overwhelmed by them.

Parenting involves many responsibilities and high-energy tasks. Yue et al. (2016) discuss the health behavior model, which seeks to explain that the physical well-being of people can be influenced in a positive way by certain personality traits (Yue et al., 2016). By applying this model to playfulness, we may expect that playfulness would result in feeling more energetic (Yue et al., 2016). In addition, some of the qualities Barnett (2007) was able to find as characteristic of a playful individual included being energetic and being active. It is possible that being a more active and energetic individual can help in keeping up with tasks involved with taking care of young children, thus lessening the burden of the work.

### 1.5. Paternal and Maternal Playfulness

Although we may expect that the trait of playfulness would be apparent in all parents, there may be differences in levels of playfulness between mothers and fathers. It is generally expected that fathers and mothers display different types of playful behaviors. For example, Majdandžić et al. (2016) stated that paternal play is typically more physical, while mothers should prefer pretend play. Paquette (2004) also theorized that fathers tend to excite, surprise, and encourage children to take more risks, whereas mother–child relationships seem calmer and more comforting. However, based on their review of 34 observational studies published from 2000 onwards, Teufl and Ahnert (2022) conclude that mothers and fathers do not differ based on the repertoire of play behavior and the play quality. Likewise, more recent studies on parent–child playful interaction find that parents are more similar than they are different in terms of effort deployed by the parent (Ahnert et al., 2017; Bureau et al., 2014), emotional availability of the parent (John et al., 2013), their support of autonomy (Olofson & Schoppe-Sullivan, 2022), and their challenging behaviors (Deneault et al., 2022; Majdandžić et al., 2016). Schmiedel (2021) also showed that mothers and fathers both use similar physical techniques (i.e., chasing and tickling), with similar success, when instructed to make their preschool children laugh. Cabrera and Roggman (2017) suggest that, in terms of being playful and engaged, fathers can be just as developmentally supportive as mothers with their children. It is also noteworthy that, contrary to popular belief, mothers tend to spend as much time playing with children as fathers (Amodia-Bidakowska et al., 2020). The redefinition of parenting roles in contemporary society may have led to greater similarities between mothers and fathers in terms of engagement and involvement with children in recent years (Cabrera et al., 2000, 2014; Fagan et al., 2014).

To further support these studies, Menashe-Grinberg and Atzaba-Poria (2017) found that mothers and fathers displayed similar levels of playfulness and that parental playfulness, regardless of gender, was associated with lower child negativity. Interestingly, the study revealed differing moderation patterns between mothers and fathers: children displayed low negativity when mothers displayed either high sensitivity, structuring, or playfulness, whereas low child negativity was observed only when fathers exhibited both high sensitivity/structuring and high playfulness (Menashe-Grinberg & Atzaba-Poria, 2017). As interpreted by the authors, this suggests that low maternal playfulness may be compensated for by other positive maternal behaviors, while paternal playfulness appears to be a necessary component for fostering low negativity in children (Menashe-Grinberg & Atzaba-Poria, 2017).

Similarly, Levavi et al. (2020) found that fathers of children with intellectual disabilities who reported lower playfulness had children with more behavioral problems, the same effect that was not observed in mothers. Finally, Shorer and Leibovich (2022) reported that higher levels of paternal playfulness were associated with lower stress reactions in children during the COVID-19 pandemic. Altogether, results suggest minimal differences in expression of playfulness between fathers and mothers; however, the pattern of association with child outcomes may differ according to parent gender.

Building on this, another interesting question is whether associations between playfulness and other areas of parenting are the same or different for each parent. A study by Proyer (2014a) found that men and women sometimes differed in the perceived functions of playfulness in daily life, even though they reported similar levels of playfulness. For example, more women than men indicated humor and laughter as a function of playfulness in leisure time, whereas more men than women reported creativity as a function of playfulness in leisure time (Proyer, 2014a). Additionally, it was found that men who reported being more playful listed more functions of playfulness, whereas the level of playfulness in women was unrelated to how many functions they listed (Proyer, 2014a). However, the results also revealed that both men and women perceived motivating oneself and others and facilitating relationships as functions of playfulness (Proyer, 2014a). These findings indicate that though levels of playfulness can be similar, men and women may not always use this trait in the same ways, or they may exhibit playfulness for different reasons. Although this study provides some context on how adult playfulness can differ, there is still a lack of understanding of how playfulness can differ for parents specifically. In an attempt to fill some of the gaps in the literature, we will be exploring the differences and similarities in the trait of playfulness between mothers and fathers.

### 1.6. The Present Study

The primary goal of the following study is to better understand the characteristics of playfulness in parents and how it may be associated with other areas of parenting. Specifically, we are interested in the associations between parental playfulness and role overload, the coparenting relationship, other parenting behaviors, and children’s playfulness. In addition, we will be exploring the differences in levels of playfulness between mothers and fathers and whether the associations between playfulness and other variables differ between parents as well. Moreover, we include measures of both trait playfulness (Adult Playfulness Scale, Glynn & Webster, 1992) and parent–child dyad-specific playfulness (*Parental Playfulness Questionnaire*, Shorer et al., 2021), with the expectation that associations will be stronger for the latter. Our objectives and hypotheses are guided by our review of the available literature on the subject.

#### 1.6.1. Objective 1: Parental Playfulness and Children’s Playfulness

The first objective (Obj. 1) is to understand the relationship between parental playfulness and children’s playfulness. Based on previous studies (von Salisch, 2001; Wu et al., 2024), which have demonstrated a positive correlation between parental playfulness and child playfulness or the potential to transmit traits through behavior, we first hypothesize (H1) a significant positive correlation between these traits.

#### 1.6.2. Objective 2: Parental Playfulness and Other Parenting Behaviors

The next objective (Obj. 2) is to explore the relationship between parental playfulness and parenting behaviors. Despite the fact that this topic remains understudied, we were able to find research pertaining to the link between playfulness or certain play behaviors and potentially positive parenting behaviors (Majdandžić et al., 2016; Román-Oyola et al., 2017). Therefore, we hypothesize (H2a) a positive correlation between parental playfulness and the frequency with which positive parenting behaviors are displayed, and (H2b) a negative relationship between parental playfulness and the frequency with which negative parenting behaviors are displayed.

#### 1.6.3. Objective 3: Parental Playfulness and Coparenting

Another objective (Obj. 3) is to investigate the relationship between parental playfulness and the coparenting relationship. Parental playfulness may be able to foster a healthier relationship between parents (Aune & Wong, 2002; Baxter, 1992; Fredrickson, 2001; Proyer, 2014b) but could also facilitate negative feelings in partners that are not as playful, which could lead to adverse effects not only for the relationship (Anderson et al., 2017; Bureau et al., 2023; McBride et al., 2005). Taking these ideas into account, and understanding that the relationship could render results in either direction, the association between parental playfulness and coparenting, in this study, will be exploratory (H3).

#### 1.6.4. Objective 4: Parental Playfulness and Role Overload

The fourth objective (Obj. 4) is to examine the association between parental playfulness and role overload. It seems that the characteristic of playfulness aids in strengthening qualities that may help with lowering stress (Shorer & Leibovich, 2022) and increasing energy (Yue et al., 2016). With these studies and assumptions in mind, we hypothesize (H4) that there will be a negative correlation between parental playfulness and role overload.

#### 1.6.5. Objective 5: Differences in Playfulness Between Mothers and Fathers

The final objective (Obj. 5) is to discover whether there are differences not only in levels of playfulness between each parent but also whether the associations between variables differ between mothers and fathers. In the past, the literature has suggested potential differences in levels of playfulness between parents (Paquette, 2004); however, more recent findings have supported the idea of mothers and fathers having a similar degree of playfulness (Bureau et al., 2014; Majdandžić et al., 2016; Teufl & Ahnert, 2022). In terms of differences between associations, the research available is minimal; however, there is some discussion about there being differences and similarities in the way that each parent views the function of playfulness (Proyer, 2014a). On the basis of the literature we discussed, we hypothesize (H5a) that there will be no significant difference in levels of playfulness between parents, and as for the differences in associations, due to the limited findings, this relationship will be exploratory (H5b).

## 2. Materials and Methods

### 2.1. Participants

This sample consisted of 348 parents of children aged 3–8 years old (*M_age_* = 61.25 months, *SD* = 16.46). Approximately half of the children were girls (52.9%), with the rest being boys (36.5%) or non-specified by the parent (10.6%). The parents consisted of 294 mothers (84.7%) and 53 fathers (15.2%). The parents ranged in age from 24 to 47 years old (*M_age_* = 35.84 years, *SD* = 4.80). The majority of the participants identified as Caucasian (76.4%); other ethnicities included were Asian (7.8%), Black (6.0%), Indigenous/Metis (0.3%), Latinx (2.9%), Middle Eastern (2.3%), other (1.1%), and mixed-race individuals (3.2%). More than half of the families reported having a gross annual income above CAD 100,000 (56.8%), and a few families reported having a gross annual income less than CAD 30,000 (6.3%). Most parents reported acquiring either an Undergraduate degree (42.8%) or a Graduate degree (27.6%) as their highest level of education. More than half of the participants completed the survey in English (75.6%), and the rest completed it in French (24.4%).

### 2.2. Procedures

We recruited participants through social media advertisements via Kijiji, Facebook, and a local newspaper, as well as by word of mouth. Participants were eligible to take part in the study if they were able to participate in French or English and had a child aged between 3 and 8 years old. Only one parent per family was able to participate in the study. Participants contacted the research team to receive an individualized link to the questionnaire package on Qualtrics, which took approximately 60 min to complete. We instructed the parents to keep one specific child in mind while they answered various questions that addressed their parenting styles, behaviors, relationships, and level of playfulness. Participants filled out consent forms, and the study was approved by the University of Ottawa Research Ethics Board.

### 2.3. Measures

#### 2.3.1. Parental Playfulness

Parental playfulness was assessed using *The Parental Playfulness Questionnaire* (PPQ; Shorer et al., 2021) and *The Adult Playfulness Scale* (APS; Glynn & Webster, 1992). The PPQ consists of 20 items scored on a 5-point Likert scale. This scale measures playfulness in parents displayed in everyday interactions with their children. Total scores from this scale are measured by averaging the scores from each item. Higher scores indicate greater levels of playfulness. The PPQ total score shows good internal consistency in the current study, α = 0.76. The APS has a total of 32 items with opposing characteristics on each end of the scale. The scale consists of 2 adjectives separated by 8 points per line. Participants are asked to circle the point closer to the characteristic that they feel they identify with more. This questionnaire is designed to measure adult playfulness within their work environment and does so using items ranging from calm to agitated, animated to passive, childlike to mature, etc. All items are reverse-scored except for predictable–unpredictable and serious–playful. Scores are averaged across items, with higher scores indicating more playfulness. The APS total score shows excellent internal consistency in the current study, α = 0.91.

#### 2.3.2. Parenting Behaviors

Parenting behaviors were measured using *The Challenging Parenting Behavior Questionnaire* (CPBQ; Majdandžić et al., 2018) and *The Parental Behaviors Questionnaire* (PBQ; Zubrick et al., 2014). The CPBQ consists of 18 items scored on a 5-point Likert scale. In the original questionnaire, items are separated into 6 different scales. For the purposes of the current study, 3 scales were retained: teasing (e.g., “I play little tricks on my child.”), rough-and-tumble play (e.g., “I enjoy tickling my child.”), and encouragement of risk-taking (e.g., “If my child finds something scary, I encourage him/her to carry on regardless.”). Scores across these scales were averaged for an overall measure of challenging parenting behavior. Higher scores indicate more challenging behaviors. The CPBQ total score shows good internal consistency in the current study, α = 0.71.

Within the PBQ, we selected the items most representative of each of the seven dimensions featured on the questionnaire: parental warmth, parental hostility, parenting anger, parenting consistency, parental separation anxiety, inductive reasoning, and parenting efficacy. The questions are scored on a 5-point Likert scale and ask the parents how often they display each behavior.

#### 2.3.3. Coparenting Relationship

Coparenting was assessed using the *Coparenting Relationship Scale* (CRS; Feinberg et al., 2012). The CRS includes 35 items rated on a 6-point Likert scale. This scale measures coparenting relationships by describing ways in which the participant and their partner parent together and asking how true each description is of the couple (e.g., “My partner and I have the same goals for our child”). Items are scored by averaging up totals within 8 subscales: coparenting agreement, coparenting closeness, exposure to conflict, coparenting support, coparenting undermining, endorsement of partner parenting, division of labor, and a brief measure of coparenting. The internal consistency scores (α) vary between 0.70 and 0.93 for the various subscales, except for division of labor with an α = 0.44. Therefore, we have chosen to exclude it.

#### 2.3.4. Parental Role and Involvement

Parental role was measured using *The Role Overload Scale* (ROS; Thiagarajan et al., 2006). The ROS measures how overloaded a person feels by the multiple roles in their life. It includes six items, which are scored on a 7-point Likert scale. Each item measures how people feel their time is spent (e.g., “I do not ever seem to have any time for myself.”). Scores are averaged, and higher scores indicate that participants feel more overloaded in their role. The ROS total score shows excellent internal consistency in the current study, α = 0.90.

#### 2.3.5. Children’s Playfulness

Children’s playfulness was assessed using *The Children’s Playfulness Scale* (CPS; Barnett, 1991b) and *The Child Behaviors Inventory of Playfulness* (CBI; Rogers et al., 1998). Both scales are composed of 23 items that are scored on a 5-point Likert scale. The CPS measures playfulness in children within five different subscales, including physical spontaneity, social spontaneity, cognitive spontaneity, manifest joy, and sense of humor. The scores are totaled by subscale. The internal consistency scores (α) vary between 0.67 and 0.75 for the various subscales. The CBI measures playfulness in children using different descriptors (e.g., “plays eagerly”) and asks the parent how characteristic this descriptor is of their child. Total scores are determined by averaging scores from each item. The CBI total score shows excellent internal consistency in the current study, α = 0.89. For both questionnaires, higher scores indicate more playfulness in children.

#### 2.3.6. Sociodemographic Information

Participants completed a standard questionnaire on socioeconomic information. For the current study, we used child age, informant age, family income, education level, and parental involvement.

### 2.4. Plan of Analysis

Associations between parental playfulness and other variables were calculated using a series of correlations followed by multiple regressions. Due to the relatively large number of associations, we will restrict our interpretation to correlations with significance thresholds of 0.01 and 0.001. As variables needed to be controlled, partial correlations were used for all the comparisons for objectives 1–4. Following this, we assessed our fifth objective by initially comparing the gender of the parent by running t-test analyses. Then the gender of the parent was used as a moderator, and a regression analysis was conducted. All analyses were conducted using the Statistical Package for the Social Sciences (SPSS, version 27).

## 3. Results

### 3.1. Preliminary Analyses

The first step in our analysis was to investigate potential effects of sociodemographic variables such as child age, child sex, informant age, family income, education level, and parental involvement. We ran correlations between the sociodemographic variables and the total scores from each questionnaire, apart from the CRS, CPS, and PBQ. Correlations between sociodemographic variables and study variables are presented in Supplementary Table S1. For the CRS and CPS, we used the total score from each subscale, and for the PBQ, we analyzed each item separately to represent each concept. Child age was significantly and positively correlated with children’s social spontaneity (r = 0.12, *p* < 0.05), cognitive spontaneity (r = 0.13, *p* < 0.05), and sense of humor (r = 0.14, *p* < 0.05). Level of education was associated with parental separation anxiety (r = 0.24, *p* < 0.01) and children’s manifestation of joy (r = 0.11, *p* < 0.05). Informant age was significantly and negatively associated with general adult playfulness (r = −0.16, *p* < 0.01) and parental inductive reasoning (r = −0.13, *p* < 0.05). A significant effect of family income was found for parental separation anxiety (r = 0.25, *p* < 0.01), inductive reasoning (r = −0.12, *p* < 0.05), and children’s manifestation of joy (r = 0.17, *p* < 0.01). Additionally, child sex was significantly associated with children’s playfulness (r = 0.12, *p* < 0.05), parental anger (r = −0.12, *p* < 0.05), coparenting support (r = 0.13, *p* < 0.05), coparenting partner parenting (r = 0.14, *p* < 0.05), and children’s physical (r = −0.24, *p* < 0.01), social (r = 0.14, *p* < 0.01), and cognitive spontaneity (r = 0.17, *p* < 0.01).

Studies have shown that the amount of time parents spend with their children can have an impact on various child outcomes (Hsin & Felfe, 2014; Fiorini & Keane, 2014; Zick et al., 2001). The amount of time parents spend with their children can also be associated with various aspects of parenting, potentially even the trait of playfulness. It is for that reason we tested the variable of parental involvement as a control variable. In doing so, we found significant associations between parental involvement and various other scores. Parental involvement was significantly correlated with parental playfulness (r = 0.30, *p* < 0.01), general adult playfulness (r = 0.25, *p* < 0.01), parental hostility (r = −0.16, *p* < 0.01), parental inductive reasoning (r = 0.13, *p* < 0.05), role overload (r = −0.14, *p* < 0.01), children’s playfulness (r = 0.25, *p* < 0.01), child’s exposure to conflict (r = −0.14, *p* < 0.01), social spontaneity (r = 0.13, *p* < 0.05), cognitive spontaneity (r = 0.19, *p* < 0.01), manifestation of joy (r = 0.24, *p* < 0.01), and sense of humor (r = 0.16, *p* < 0.01). These findings indicate that parents who report being more involved report themselves as being more playful, as well as their child being more playful, more socially and cognitively spontaneous, having more of a sense of humor, and manifesting joy. Additionally, higher parental involvement was associated with less exposure to conflict and feelings of being overloaded by their role. Moreover, parental involvement was found to be negatively correlated with parental hostility and anger. Considering the patterns we found, we controlled for specific variables when they were involved (see tables for details).

### 3.2. Primary Analyses

#### 3.2.1. Objective 1: Children’s Playfulness

As expected, the results of the partial correlation analysis using the Parental Playfulness Questionnaire (PPQ), Adult Playfulness Scale (APS), Children’s Playfulness Scale (CPS), and Child Behavior Inventory (CBI) indicated that parental playfulness and adult playfulness were positively associated with several dimensions of child playfulness (Table 1). Significant associations were found between parental playfulness and child’s manifestation of joy (r = 0.29, *p* < 0.001), sense of humor (r = 0.28, *p* < 0.001), cognitive spontaneity (r = 0.37, *p* < 0.001), and the child’s behavior inventory (r = 0.36, *p* < 0.001). The multiple regression showed that the child playfulness dimension explained, altogether, a significant portion of variance in parental playfulness after controlling for sociodemographic variables: ΔF(6, 268) = 11.43, *p* < 0.001. More specifically, a child’s manifestation of joy (β = 0.15, *p* = 0.019), sense of humor (β = 0.19, *p* = 0.001), and cognitive spontaneity (β = 0.20, *p* = 0.005) remain significant predictors when shared variance is taken into account.

Significant correlations were also found between general adult playfulness and sense of humor (r = 0.15, *p* < 0.01). However, the results of the multiple regression showed that the child’s playful dimensions did not predict the general adult playfulness altogether, ΔF(6, 267) = 1.497, *p* = 0.07.

#### 3.2.2. Objective 2: Parental Behaviors

Partial correlation analyses were run for associations between parental and adult playfulness and various parenting behaviors (Table 2). Parental and adult playfulness were both positively correlated with challenging parenting behaviors (PPQ: r = 0.15, *p* < 0.01; APS: r = 0.16, *p* < 0.01). Only parental playfulness was significantly associated with parental warmth (r = 0.21, *p* < 0.001), parental hostility (r = −0.19, *p* < 0.001), parental anger (r = −0.21, *p* < 0.001), and parental efficacy behaviors (r = 0.18, *p* < 0.001). After controlling for sociodemographic variables, multiple regression analyses indicated that parenting behaviors explained a significant portion of variance in parental playfulness: ΔF(8, 265) = 4.11, *p* < 0.001. Specifically, challenging parenting behaviors (β = 0.12, *p* = 0.032) and warm parenting behaviors (β = 0.18, *p* = 0.002) remained significant predictors. However, when exploring parenting behaviors in predicting general adult playfulness, the multiple regression analyses rendered insignificant results: ΔF(8, 263) = 1.55, *p* = 0.141. Although, specifically, challenging parenting behaviors (β = 0.13, *p* = 0.026) remained a significant predictor.

#### 3.2.3. Objective 3: Coparenting Relationship

Partial correlations were conducted for relationships between parental playfulness and adult playfulness, and the coparenting subscales (Table 3). Unlike the aforementioned variables, the coparenting relationship was not associated with adult playfulness on any of the subscales. Parental playfulness was significantly correlated with coparent undermining (r = −0.23, *p* = 0.01) and endorsing partner parenting (r = 0.18, *p* < 0.01). Multiple regression analyses were conducted and indicated that the coparenting relationship explained a significant portion of variance in parental playfulness: ΔF(7, 242) = 3.69, *p* < 0.001. Particularly, coparent undermining (β = −0.15, *p* = 0.025) and endorsing partner parenting (β = 0.16, *p* = 0.016) remained significant predictors, with the addition of coparent closeness (β = −0.14, *p* = 0.04).

#### 3.2.4. Objective 4: Role Overload

After running a partial correlation between parental playfulness and role overload, controlling for parental involvement, we did not find any significant associations (r = −0.046, *p* = 0.40). Likewise, there were no significant correlations between role overload and general adult playfulness (r = −0.046, *p* = 0.41). We also explored the association between role overload and hours spent outside the home in an attempt to understand if there was an impact on how overloaded one is, not only by parenting, but by activities outside of the home as well. The relationship was found to be insignificant (r = 0.05, *p* = 0.39).

#### 3.2.5. Objective 5: Mother–Father Differences

To understand the differences between mothers and fathers in the context of playfulness, we conducted a regression analysis to discover moderation effects. First, we explored whether there was a difference between mother and father playfulness independently, before examining the differences in playfulness within other parenting areas. We conducted t-test analyses to compare the two groups, and we found the following significant differences between mothers’ and fathers’ mean scores: coparenting undermining (M_mom_ = 2.56, SD_mom_ = 1.65; M_dad_ = 3.63, SD_dad_ = 1.92; t = −3.56, *p* < 0.001), child’s behavior inventory (M_mom_ = 4.07, SD_mom_ = 0.52; M_dad_ = 3.85, SD_dad_ = 0.48; t = 2.85, *p* = 0.005), child’s manifestation of joy (M_mom_ = 4.48, SD_mom_ = 0.48; M_dad_ = 4.21, SD_dad_ = 0.62; t = 2.93, *p* = 0.005), child’s sense of humor (M_mom_ = 3.84, SD_mom_ = 0.75; M_dad_ = 3.48, SD_dad_ = 0.70; t = 3.16, *p* = 0.002), child’s cognitive spontaneity (M_mom_ = 4.03, SD_mom_ = 0.83; M_dad_ = 3.57, SD_dad_ = 0.84; t = 3.67, *p* < 0.001), and challenging parenting behaviors (M_mom_ = 3.35, SD_mom_ = 0.59; M_dad_ = 3.66, SD_dad_ = 0.74; t = −2.93, *p* = 0.005).

**Figure 1:**
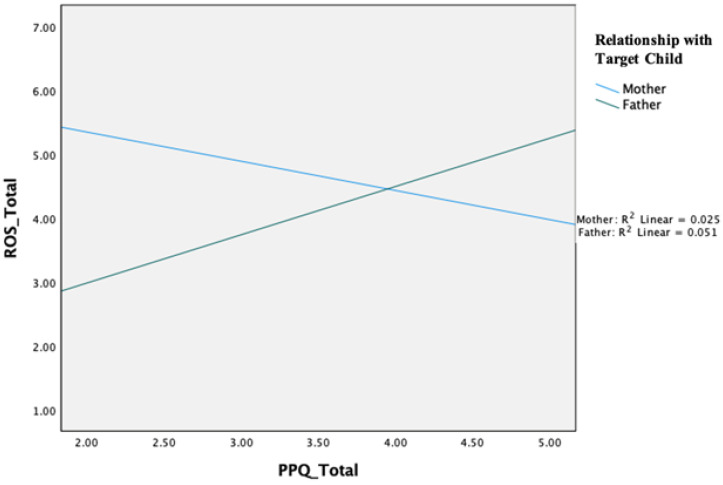
Objective 5: Moderation effect of parental gender on role overload.

A hierarchical linear regression analysis, testing a potential moderating effect of parental gender, first showed that parental involvement significantly predicted role overload: ΔF(1, 333) = 6.02, *p* = 0.015, β = −0.13, *p* = 0.015. The analysis also showed a significant main effect for parental gender, ΔF(2, 331) = 4.42, *p* = 0.013, β = −0.15, *p* = 0.005, suggesting that mothers report being less overloaded. Although there was not a significant main effect of parental playfulness, we were able to find a significant interaction term in the prediction of role overload: ΔF(1, 330) = 6.83, *p* = 0.009, β = 1.24, *p* = 0.009 (Figure 1). To explore that moderation effect, we explored the associations between parental playfulness and role overload separately for each gender. This analysis revealed a negative association for mothers (r = −0.16, *p* = 0.007), whereas the association was positive for fathers (r = 0.23, *p* = 0.11). None of the other regression models revealed significant moderation effects.

## 4. Discussion

The present study sought to investigate associations between parental playfulness, child’s playfulness, and other areas of parenting. As hypothesized (H1) we found positive associations between parental and children’s playfulness. We also found positive associations between parental playfulness and challenging and warmth parenting behaviors as well as parental efficacy (H2a) and negative associations between parental playfulness and parental hostility and anger (H2b). In addition, we found that higher parental playfulness was associated with higher endorsement of partner parenting and lower partner undermining (H3). Contrary to our hypothesis, we have not observed a significant association between parental playfulness and role overload (H4). Finally, we found some significant differences between mothers and fathers in specific areas of parenting, but not playfulness (H5a), and a moderation of parent gender on the association between playfulness and role overload (H5b).

### 4.1. Objective 1: Children’s Playfulness

As hypothesized, playfulness in parents is significantly related to most areas of children’s playfulness. Moreover, specific dimensions of children’s playfulness were found to be significantly associated with parental playfulness. Our results are in line with previous research that found that young children learned the trait of playfulness through imitation of their parents (Youell, 2008). Interestingly, adult playfulness (a more general dimension) and parental playfulness (a more specific dimension) differed in the way they related to children’s playfulness. There were more significant associations between children’s playfulness and parental playfulness than with general adult playfulness. Additionally, the regression analyses indicated child dimensions were significant predictors for parental playfulness, whereas the same was not found for general adult playfulness. Greater proximity to and complicity with a child, in general, or having a more playful child may incite playfulness in parents that are not necessarily playful in other dimensions of their life (e.g., work, friends, romantic relationships) and when interacting with other people. It could also be possible that if parents report being more playful, they may feel obligated to also report their child as more playful. More research should be performed to disentangle how and why adults tend to be playful and in which contexts this playfulness can be observed.

Additionally, we found that the dimensions which rendered significant associations with children’s playfulness and remained as significant predictors of parental playfulness fell under the cognitive and emotional aspects of playfulness (e.g., cognitive spontaneity and sense of humor) as opposed to the physical and social aspects. This may suggest that a playful parent may influence only certain aspects of child playfulness, whereas peers may influence other aspects. Being “quick with a joke” or understanding humor requires advanced cognitive abilities that may be taught by parents. For example, a parent may explain that what they just said was sarcasm, or they may explain concepts such as second degree. Peers of the same age may be less likely to be able to teach such concepts or even understand them. Parents may also have a greater impact on the level of exuberance they tolerate at home, which could explain an association with the manifestation of joy. However, parents may have less influence on their child’s popularity amongst their peers, or whether they prefer physical play over other types of play, concepts that may be more related to interactions with peers.

Unfortunately, many of the previous studies on child playfulness exclusively focused on teachers’ or parents’ observations of child physical play with peers (see Trevlas et al., 2003), which is only one of the many settings in which child playfulness can be expressed. Nonetheless, a few studies linked child playfulness to concepts such as emotional adjustment (Barnett, 1991a) and emotion coping techniques (Saunders et al., 1999), therefore supporting the importance of the cognitive and emotional aspects of child playfulness.

### 4.2. Objective 2: Parental Behaviors

The association between playfulness and other parenting behaviors was hypothesized to be positive between parental playfulness and the frequency of positive parenting behavior and negative between parental playfulness and the frequency of negative parenting behaviors. Although challenging a child may be seen as insensitive by some, Majdandžić et al. (2016) instead found that fathers who challenged their child in play also tend to be warmer in their interaction with them. Our results support this previous observation as we were able to find that playfulness in parents was associated with both challenging parenting behaviors and warm parenting behaviors, indicating that playful parents may push their children out of their comfort zones but also express affection and provide support. In addition, our results indicated that these specific parenting behaviors were significant predictors of parental playfulness. Challenging parenting behaviors without warmth could potentially overlap with intrusiveness; thus, the co-occurrence of both behaviors may be what makes it healthy for development. Such a proposition is in line with previous results by Hazen et al. (2010) showing that children whose fathers presented both frightening behaviors and sensitivity while playing with them in infancy had better regulation skills at 24 months and 7 years of age. In comparison, children whose fathers showed only frightening behaviors but low sensitivity displayed more emotion regulation problems during their development.

In addition, another study indicated that adult playfulness was a relevant predictor of emotional parental self-efficacy (Román-Oyola et al., 2017). Likewise, our findings revealed a significant association between parental efficacy and parental playfulness, potentially indicating that playful parents feel that they have more control and a greater ability to effectively parent their child. We could also conceive of the reverse. When a parent feels efficient in their role, they may be less worried and indecisive, thus allowing them the space to be more playful in their interactions.

Our results indicated a negative relationship between parental playfulness and hostility and anger. This is consistent with past studies and our hypothesis. A study conducted by Chang et al. (2013) found that the trait of playfulness was associated with more positive emotions and fewer negative emotions. Similarly, Shorer et al. (2021) found that higher levels of parental playfulness were positively related to parents’ emotional awareness. Playfulness has also been linked to better coping methods (Magnuson & Barnett, 2013), thus making certain situations easier to handle. Moreover, many people are moving in the direction of solving things with positive parenting, which may be generally changing the way parents respond to their children’s behavior (Durrant, 2020). As this is correlational, we must also consider that playful parents create an environment where they do not need to exhibit hostile or angry behavior towards their children.

While parenting variables such as efficacy, anger, and hostility were significantly associated with parental playfulness at the correlational level, it is important to note that our regression analysis only found warm and challenging behaviors to uniquely predict parental playfulness when accounting for shared variance. This may suggest that although traits such as feeling effective in parenting or being less angry may be related to being playful, they may not be such prominent contributing factors.

Finally, we found that parenting behaviors are associated in the context of parental playfulness and not just playfulness in general. This brings some attention to the validity of the concept that there is something about being a playful parent and not just being playful generally. Furthermore, we did not find parenting behaviors to be significant in predicting general adult playfulness altogether.

### 4.3. Objective 3: Coparenting Relationship

Due to conflicting ideas on the way that coparenting could be related to playfulness, our analysis was exploratory. The results of this study revealed a significant positive association between parental playfulness and endorsing partner parenting. The results also revealed a significant negative association with parental playfulness and coparenting undermining. In addition, our regression analyses revealed that higher endorsement of partner parenting and lower partner undermining were significant predictors of parental playfulness. Playful parents may be encouraged to show playful behaviors more when they feel supported by their partner. This would be consistent with the Spillover hypothesis (Erel & Burman, 1995), stating that parents are more engaged in positive interaction with children if they feel supported in their relationship with their partner. It is also possible that playful parents are more involved and foster more positive interactions with their child, thus encouraging their partner to be more supportive. Similarly, partners may appreciate playfulness in a partner, thus resulting in less undermining, or if a partner is less undermining, it may give the other parent space to be more playful. Likewise, in the case of the association between coparenting and partner parenting, either a more playful parent may interpret more positive partner parenting, or if a partner positively parents, it may give room for more playfulness.

Interestingly, our regression analysis revealed that lower coparent closeness was found to be a significant predictor of parental playfulness. This association should be interpreted with caution as it only appears significant when combined with other predictors. Nonetheless, this may suggest that playfulness may trigger jealousy and resentment at least in some couples. According to our results, it seems as though the trait of playfulness can contribute positively to the coparenting relationship; however, due to the correlational nature of the study, our suggestions are speculative.

### 4.4. Objective 4: Role Overload

Contrary to what was hypothesized, role overload was not significantly associated with parental or adult playfulness. The assumption that playfulness and role overload would be negatively correlated was supported by the idea that playful individuals may have more effective coping strategies and may be better able to manage stress (Labrell, 1994; Saunders et al., 1999; Shorer & Leibovich, 2022). In addition, we assumed that as playfulness results in higher energy levels, it may be able to mediate high-energy parenting tasks (Barnett, 2007; Yue et al., 2016). Our results, however, potentially indicate that the burden of parenting is not related to whether a parent is playful or not. As this study was conducted during the COVID-19 pandemic, parenting load may have been increased due to extra time spent playing, caring for, and potentially homeschooling children (Prime et al., 2020). Therefore, factors other than playfulness may have affected parents’ stress and role-overload during these stranger times.

Furthermore, the relation between playfulness and being overloaded by one’s role as a parent can differ between parents. Some parents may use playfulness as a means to alleviate stress and, thus, feel less overwhelmed. Conversely, playfulness for some parents can be an indicator of greater involvement, thereby influencing the feelings of being overwhelmed. More research should be conducted to fully understand the relationship between these two variables.

### 4.5. Objective 5: Mother–Father Differences

According to the recent studies addressing differences in playfulness in mothers and fathers, we hypothesized finding no differences in levels of playfulness between parents. In line with our prediction, when comparing the two groups, we were unable to find significant differences in the level of playfulness within them. We found that parents differed in six other areas, and only one of them was related to parental playful behavior directly: challenging behaviors. The general lack of difference in parental playfulness is in line with recent meta-analytic results suggesting that fathers and mothers do not differ in terms of play behaviors (Teufl & Ahnert, 2022). However, the results showing that fathers reported using more challenging behaviors than mothers tend to support the activation theory (Paquette, 2004). Additionally, we were able to find a moderation effect of playfulness on gender for the variable of role overload. This analysis showed that parental playfulness was positively associated with role overload for fathers, but this relation was negative for mothers. Mothers are still involved and are sometimes seen as the primary caregivers for children (Pakaluk & Price, 2020). This could indicate that, as they are already so involved, adopting a playful approach or attitude to parenting may alleviate the feeling of being overloaded. Conversely, the father’s role is less socially prescribed (Cabrera et al., 2000); thus, their level of playfulness may be an indicator of their greater involvement with children and other aspects of parenting, leading to a feeling of being overwhelmed.

### 4.6. Limitations

Despite our ability to conduct various analyses and find significant associations between playfulness and different areas of parenting, our study is not without limitations. First, our study contains only correlation and regression analyses, meaning that although we can find significant relationships, we cannot assume causation between variables. Using longitudinal design testing for reciprocal associations (e.g., RI-CLPM analysis) can assist in clarifying the directionality between variables (Ahn et al., 2022).

Second, only one informant completed all of the questionnaires. If the parent perceives themselves to be more playful, they may exaggerate their answers on other questionnaires to correspond with this trait. Ideally, having a second parent or an observer report on these traits would allow for more than one perspective and add to the objectivity of the measurement of variables, particularly the child’s playfulness or the coparenting relationship. Moreover, research has shown that social desirability is particularly influential in studies involving parental self-reports of their own parental behavior, as parents may feel pressure to conform to societal expectations of good parenting (Caputo, 2017; Van Ijzendoorn et al., 2004). Going forward, the use of interviews and observational methods may be beneficial to identify how playfulness is not only associated with parenting but also how it affects different areas of parenting.

Third, our sample was low-risk and included individuals who were primarily of Caucasian descent, were mothers, and were heterosexual. Future studies should look into a more ethnically diverse sample, as well as same-sex couples or families wherein fathers are the primary caregivers.

Lastly, due to the COVID-19 pandemic potentially affecting parents’ level of involvement with their children, our results may have been impacted. Parents spent more time at home with their children, but we are unsure how this may have affected playfulness in general, as some parents may have used this opportunity to spend more quality time with children, while others may have experienced more stress, worries, and mental health challenges, interfering with the quality of the relationship with their children.

## 5. Conclusions

Many studies regarding parents and families focus on how parents play with their children but fail to acknowledge the trait of playfulness and how that plays a role in parenting behaviors. Investigating playfulness has contributed to our understanding of the importance of well-roundedness in parenting. Our findings suggest that parental playfulness is a promising area for future research, going beyond traditionally studied parental behaviors. Longitudinal study designs with multiple informants and observational measures will be critical in moving playfulness research to the next step. Additionally, future research should investigate the direction of the associations between playfulness and areas of parenting and attempt to answer the following question: “are playful parents generally more positive in their interactions and in terms of parenting outcomes or does the fact that they are playful change the nature of their environment?” These studies will help set the ground for potential interventions that focus on positive behaviors to promote in parents.

## Figures and Tables

**Table 1:** Objective 1: Partial correlations between parental playfulness and child’s playfulness.

Variable		CPS Phys Spontaneity ^a,d^	CPS Manifest Joy ^a,c,f^	CPS Sense of Humor ^a,e^	CPS Soc Spontaneity ^d,e^	CPS Cog Spontaneity ^a,d,e^	CBI ^a^
PPQ ^a^	Correlation	0.04	0.29 ***	0.28 ***	0.12	0.37 ***	0.36 ***
	Significance	0.46	<0.001	<0.001	0.04	<0.001	<0.001
APS ^a,b^	Correlation	0.14	0.12	0.15 **	0.12	0.09	0.11
	Significance	0.02	0.03	0.009	0.06	0.16	0.07

Note. Controlling for ^a^ = PIQ, ^b^ = informant age, ^c^ = family income, ^d^ = child sex, ^e^ = child age, ^f^ = highest diploma. *p* < 0.01 **, *p* < 0.001 ***.

**Table 2:** Objective 2: Partial correlations between parental playfulness and other parenting behaviors.

Variable		CPBQ	PBQ Warmth	PBQ Hostility ^a^	PBQ Anger ^e^	PBQ Consistency	PBQ Parental Separation Anxiety ^c,d^	PBQ Inductive Reasoning ^b,c^	PBQ Efficacy
PPQ ^a^	Correlation	0.15 **	0.21 ***	−0.19 ***	−0.21 ***	0.09	−0.03	−0.01	0.18 ***
	Significance	0.006	<0.001	<0.001	<0.001	0.10	0.57	0.85	0.001
APS ^a,b^	Correlation	0.16 **	0.08	−0.07	−0.03	0.03	−0.06	−0.01	0.06
	Significance	0.005	0.16	0.20	0.59	0.56	0.32	0.82	0.27

Note. Controlling for ^a^ = PIQ, ^b^ = informant age, ^c^ = family income, ^d^ = highest diploma, ^e^ = child sex. *p* < 0.01 **, *p* < 0.001 ***.

**Table 3:** Objective 3: Partial correlations between parental playfulness and coparenting relationship.

Variable		CRS Cop. Agreement	CRS Cop. Closeness	CRS Exp. Conflict ^a^	CRS Cop. Support ^c^	CRS Cop. Undermining	CRS Partner Parenting ^c^	CRS Coparenting Total
PPQ ^a^	Correlation	0.09	−0.07	−0.12	0.11	−0.23 **	0.18 **	0.09
	Significance	0.14	0.26	0.04	0.07	0.01	0.003	0.14
APS ^a,b^	Correlation	0.05	−0.05	−0.12	0.06	−0.05	0.06	0.03
	Significance	0.41	0.42	0.04	0.35	0.43	0.38	0.61

Note. Controlling for ^a^ = PIQ, ^b^ = informant age, ^c^ = child sex. *p* < 0.01 **.

## Data Availability

The data file will be available via the Open Science Framework: https://osf.io/e6t32/?view_only=c18ee672dded4694af58d2e5f24990ad (accessed on 2 May 2025).

## Supplementary Materials

The following supporting information can be downloaded at: https://www.mdpi.com/article/10.3390/bs15070867/s1, Table S1: Associations between potential control variables and study variables.
