# Supplementary material for: The Associations Between Parental Playfulness, Parenting Styles, the Coparenting Relationship and Child Playfulness
Source: Behav Sci (Basel). 2025 Jun 26;15(7):867. doi: 10.3390/bs15070867 (PMC12292744; doi:10.3390/bs15070867)
Supplement: Supplementary file 1 [file behavsci-15-00867-s001.zip › behavsci-3667850-supplementary.pdf]

Supplementary Table S1. *Associations between potential control variables and study variables*

	Variable	1	2	3	4	5	6	7	8	9	10	11	12	13	14	15	16
**1.**	Family Income	-	-.03	.30**	.45**	-.20**	-.01	-.04	-.10	.01	.03	.01	-.03	.17**	-.03	.00	.13*
**2.**	Child Age	-.03	-	.20**	-.03	-.07	.07	-.09	.04	.12*	.13*	.02	.14*	-.06	-.01	.07	.01
**3.**	Informant Age	.30**	.20**	-	.28**	-.15**	.17**	-.11	-.18**	.08	.03	.02	-.05	.07	.01	-.11	-.06
**4.**	Highest Diploma	.45**	-.03	.28**	-	-.13*	.01	-.04	-.11	.03	.03	.02	-.09	.11*	-.02	-.04	.13*
**5.**	PIQ Hours Total	-.20**	-.07	-.15**	-.13	-	.04	.30**	.25**	.13*	.19**	.10	.16**	.24**	.25**	.05	.03
**6.**	Child Sex	-.01	.07	.17**	.01	.04	-	.01	.05	.14*	.17**	-.24**	.08	.07	.12*	.01	-.09
**7.**	PPQ Total	-.04	-.09	-.11	-.04	.30**	.01	-	.44**	.12*	.37**	.07	.30**	.35**	.41**	.15**	.03
**8.**	APS Total	-.10	.04	-.18**	-.11	.25**	.05	.44**	-	.16**	.18**	.16**	.20**	.16**	.24**	.17**	.00
**9.**	CPS Soc. Spontaneity	.01	.12*	.08	.03	.13*	.14*	.12*	.16**	-	.30**	.28**	.34**	.32**	.32**	-.01	.08
**10.**	CPS Cog. Spontaneity	.03	.13*	.03	.03	.19**	.17**	.37**	.18**	.30**	-	.15**	.35**	.36**	.66**	.14*	.01
**11.**	CPS Phys. Spontaneity	.01	.02	.02	.02	.10	-.24**	.07	.16**	.28**	.15**	-	.16**	.29**	.24**	.08	.12*
**12.**	CPS Sense of Humour	-.03	.14*	-.05	-.09	.16**	.08	.30**	.20**	.34**	.35**	.16**	-	.31**	.34**	.34**	.17**
**13.**	CPS Manifest Joy	.17**	-.06	.07	.11*	.24**	.07	.35**	.16**	.32**	.36**	.29**	.31**	-	.42**	.07	.19**
**14.**	CBI Total	-.03	-.01	.01	-.02	.25**	.12*	.41**	.24**	.32**	.66**	.24**	.34**	.42**	-	.05	.02
**15.**	CPBQ Total	.00	.07	-.11	-.04	.05	.01	.15**	.17**	-.01	.14*	.08	.34**	.07	.05	-	.11
**16.**	PBQ Total	.13*	.01	-.06	.13*	.03	-.09	.03	.00	.08	.01	.12*	.17**	.19**	.02	.11	-
**17.**	PBQ Warmth	.03	-.04	-.01	-.05	.10	-.07	.23**	.12*	.02	.08	.16**	.18**	.23**	.09	.08	.34**
**18.**	PBQ Hostility	-.04	.02	.03	.03	-.16**	-.06	-.23**	-.13*	-.09	-.01	.02	.03	-.01	-.08	-.01	.31**
**19.**	PBQ Consistency	.07	.05	.00	.07	.09	.03	.12*	.07	.11	.08	.02	.01	.19**	.10	.04	.38**
**20.**	PBQ Efficacy	.02	-.01	.00	.02	.08	.02	.20**	.09	.14**	.03	.01	.13*	.16**	.15**	.04	.35**
**21.**	PBQ Sep. Anxiety	.25**	-.03	.02	.24**	-.10	-.04	-.07	-.10	.05	-.11*	-.03	-.05	.01	-.12*	-.05	.60**
**22.**	PBQ Inductive Reason	-.12*	.06	-.13*	-.04	.13*	-.01	.06	.06	.07	.11*	.09	.24**	.11*	.06	.21**	.49**
**23.**	PBQ Anger	.03	.00	-.09	-.02	-.01	-.12*	-.18**	-.06	-.12*	-.09	.11*	.00	-.11	-.10	.02	.19**
**24.**	CRS Cop. Agreement	.05	.02	.04	.10	.03	.04	.09	.05	.04	.05	.04	.01	.09	.10	-.06	.20**
**25.**	CRS Cop. Closeness	-.07	.06	.03	-.04	-.01	.06	-.04	-.04	-.05	.06	.01	-.01	.02	.07	.05	.03
**26.**	CRS Exp. Conflict	.08	.01	.01	-.01	-.14*	.02	-.15**	-.13*	-.03	-.04	.00	-.08	-.07	-.04	.07	-.02
**27.**	CRS Cop. Undermining	-.05	-.05	.02	-.01	-.06	-.05	-.24**	-.08	-.10	-.14*	-.07	-.14*	-.24**	-.09	.11	-.24**
**28.**	CRS Coparenting Total	.05	-.07	.00	.05	-.08	.01	.05	.02	-.01	-.01	.02	-.06	.01	.06	.02	.14*
**29.**	CRS Cop. Support	-.04	-.01	-.03	.01	.07	.13*	.14*	.11	-.07	.01	-.08	.07	.02	.06	.09	.11
**30.**	CRS Partner Parenting	.03	-.01	.01	-.06	-.01	.14*	.17**	.06	-.06	.06	-.05	.02	.09	.08	.07	.01
**31.**	ROS Total	.04	-.04	.04	.05	-.14**	-.08	-.09	-.09	-.06	-.02	-.03	-.10	-.01	-.06	.02	-.08

$p < 0.05$*, $p < 0.01$**

Supplementary Table S1. Cont'd

	Variable	17	18	19	20	21	22	23	24	25	26	27	28	29	30	31
1.	Family Income	.03	-.04	.07	.02	.25**	-.12*	.03	.05	-.07	.08	-.05	.05	-.04	.03	.04
2.	Child Age	-.04	.02	.05	-.01	-.03	.06	.00	.02	.06	.01	-.05	-.07	-.01	-.01	-.04
3.	Informant Age	-.01	.03	.00	.00	.02	-.13*	-.09	.04	.03	.01	.02	.00	-.03	.01	.04
4.	Highest Diploma	-.05	.03	.07	.02	.24**	-.04	-.02	.10	-.04	-.01	-.01	.05	.01	-.06	.05
5.	PIQ Hours Total	.10	-.16**	.09	.08	-.10	.13*	-.01	.03	-.01	-.14*	-.06	-.08	.07	-.01	-.14**
6.	Child Sex	-.07	-.06	.03	.02	-.04	-.01	-.12*	.04	.06	.02	-.05	.01	.13*	.14*	-.08
7.	PPQ Total	.23**	-.23**	.12*	.20**	-.07	.06	-.18**	.09	-.04	-.15**	-.24**	.05	.14*	.17**	-.09
8.	APS Total	.12*	-.13*	.07	.09	-.10	.06	-.06	.05	-.04	-.13*	-.08	.02	.11	.06	-.09
9.	CPS Soc. Spontaneity	.02	-.09	.11	.14**	.05	.07	-.12*	.04	-.05	-.03	-.10	-.01	-.07	-.06	-.06
10.	CPS Cog. Spontaneity	.08	-.01	.08	.03	-.11*	.11*	-.09	.05	.06	-.04	-.14*	-.01	.01	.06	-.02
11.	CPS Phys. Spontaneity	.16**	.02	.02	.01	-.03	.09	.11*	.04	.01	.00	-.07	.02	-.08	-.05	-.03
12.	CPS Sense of Humour	.18**	.03	.01	.13*	-.05	.24**	.00	.01	-.01	-.08	-.14*	-.06	.07	.02	-.10
13.	CPS Manifest Joy	.23**	-.01	.19**	.16**	.01	.11*	-.11	.09	.02	-.07	-.24**	.01	.02	.09	-.01
14.	CBI Total	.09	-.08	.10	.15**	-.12*	.06	-.10	.10	.07	-.04	-.09	.06	.06	.08	-.06
15.	CPBQ Total	.08	-.01	.04	.04	-.05	.21**	.02	-.06	.05	.07	.11	.02	.09	.07	.02
16.	PBQ Total	.34**	.31**	.38**	.35**	.60**	.49**	.19**	.20**	.03	-.02	-.24**	.14*	.11	.01	-.08
17.	PBQ Warmth	-	-.03	-.03	.16**	-.02	.22**	-.11*	.10	.01	-.02	-.12*	.11	.00	.05	-.05
18.	PBQ Hostility	-.03	-	-.16**	-.26**	-.01	.08	.44**	-.01	.06	.16**	.05	.14*	.09	-.02	.23**
19.	PBQ Consistency	-.03	-.16**	-	.20**	.17**	.04	-.37**	.29**	.10	-.16**	-.26**	.00	.07	.13	-.28**
20.	PBQ Efficacy	.16**	-.26**	.20**	-	.08	.15**	-.41**	.14*	.04	-.12*	-.13*	.11	.14*	.06	-.30**
21.	PBQ Sep. Anxiety	-.02	-.01	.17**	.08	-	-.03	.01	.12*	-.01	-.03	-.10	.06	-.03	-.03	-.07
22.	PBQ Inductive Reason	.22**	.08	.04	.15**	-.03	-	.01	.03	-.03	-.07	-.19**	.00	.08	-.05	-.03
23.	PBQ Anger	-.11*	.44**	-.37**	-.41**	.01	.01	-	-.17**	-.06	.22**	.12*	.00	-.05	-.11	.34**
24.	CRS Cop. Agreement	.10	-.01	.29**	.14*	.12*	.03	-.17**	-	.21**	-.27**	-.44**	.46**	.30**	.26**	-.20**
25.	CRS Cop. Closeness	.01	.06	.10	.04	-.01	-.03	-.06	.21**	-	-.13*	-.05	.47**	.29**	.24**	-.11
26.	CRS Exp. Conflict	-.02	.16**	-.16**	-.12*	-.03	-.07	.22**	-.27**	-.13*	-	.29**	.05	-.19**	-.18**	.24**
27.	CRS Cop. Undermining	-.12*	.05	-.26**	-.13*	-.10	-.19**	.12*	-.44**	-.05	.29**	-	-.13*	-.10	-.23**	.15**
28.	CRS Coparenting Total	.11	.14*	.00	.11	.06	.00	.00	.46**	.47**	.05	-.13*	-	.51**	.35**	-.07
29.	CRS Cop. Support	.00	.09	.07	.14*	-.03	.08	-.05	.30**	.29**	-.19**	-.10	.51**	-	.27**	-.11
30.	CRS Partner Parenting	.05	-.02	.13*	.06	-.03	-.05	-.11	.26**	.24**	-.18**	-.23**	.35**	.27**	-	-.10
31.	ROS Total	-.05	.23**	-.28**	-.30**	-.07	-.03	.34**	-.20**	-.11	.24**	.15**	-.07	-.11	-.10	-

$p < 0.05$*, $p < 0.01$**
